# Supplementary figures and images for: Lipid-driven Src self-association modulates its transformation capacity
Source: Life Sci Alliance. 2025 Mar 13;8(5):e202403019. doi: 10.26508/lsa.202403019 (PMC11909415; doi:10.26508/lsa.202403019)

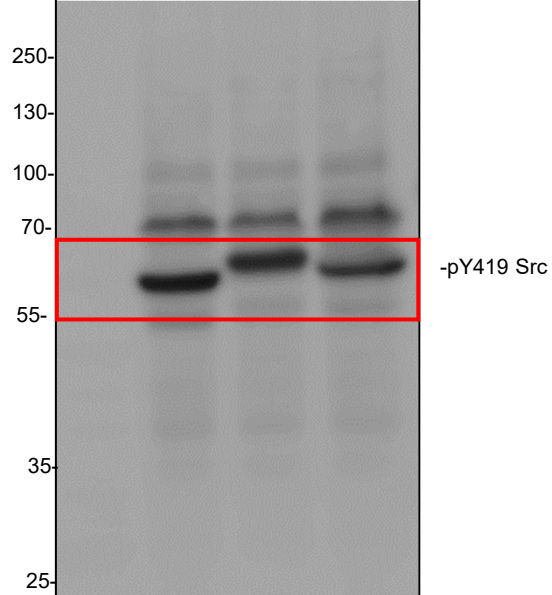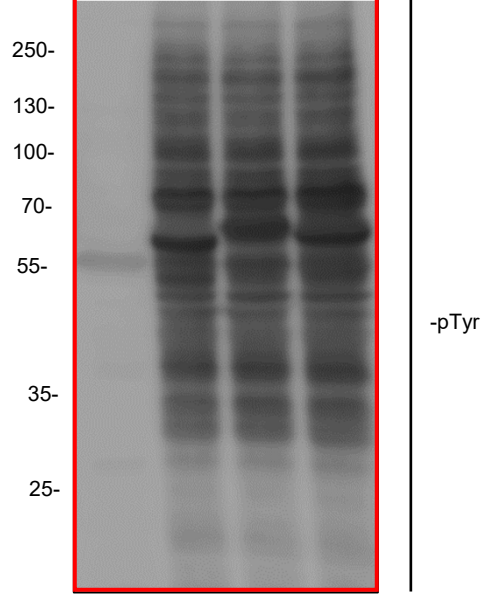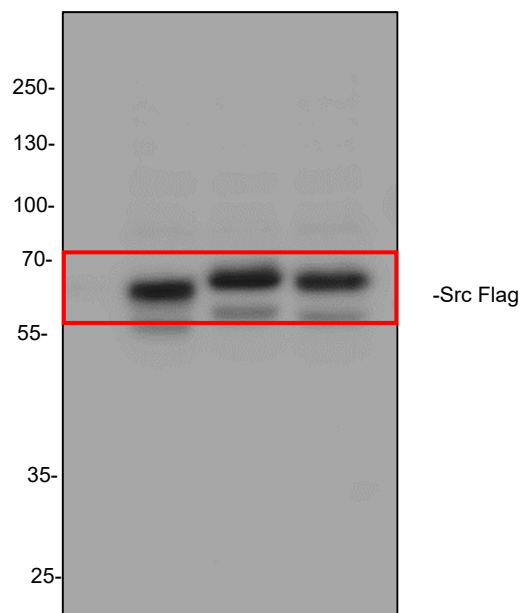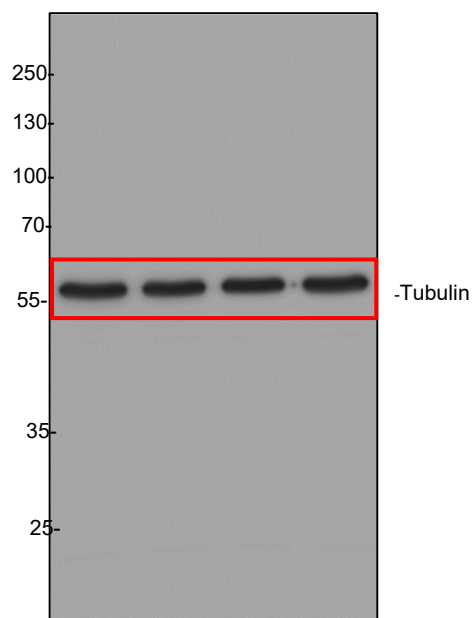

Supplement: Supplementary file 1 [file LSA-2024-03019_SdataFS4.pdf]

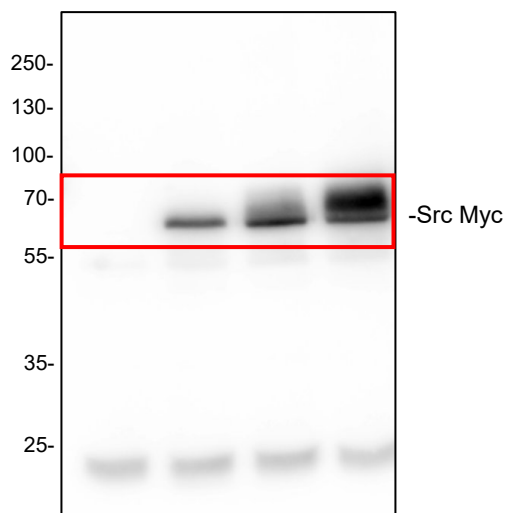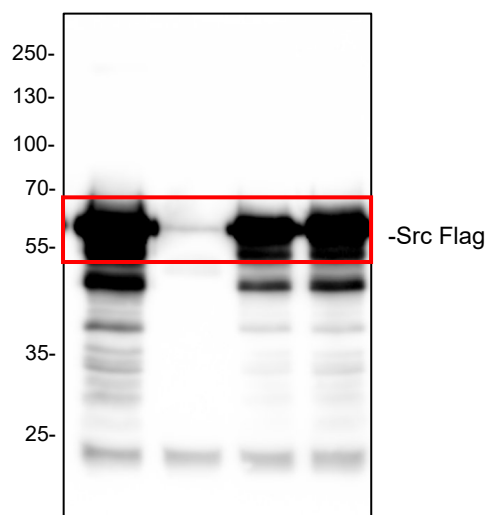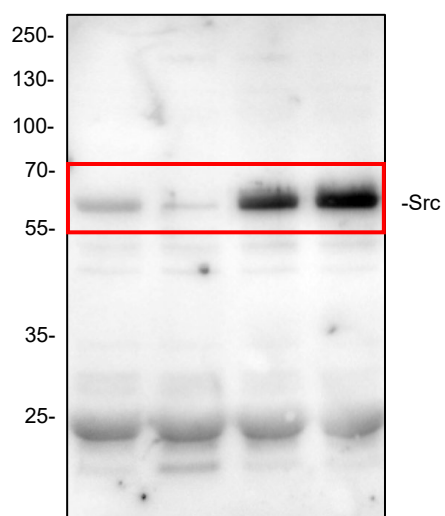

WCL

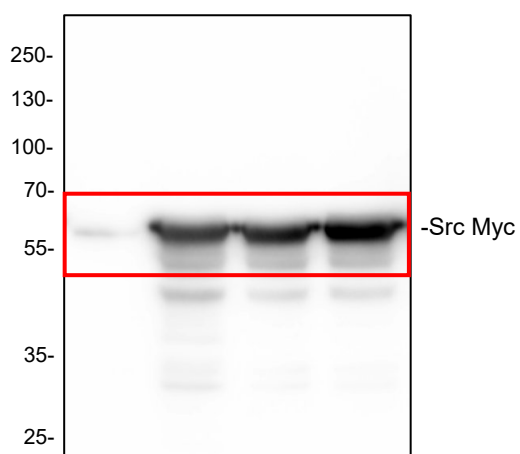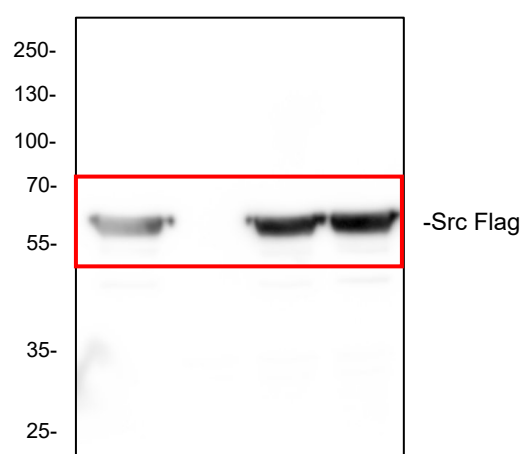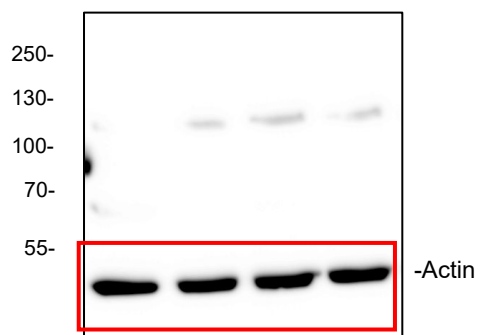

Supplement: Supplementary file 2 [file LSA-2024-03019_SdataF4.pdf]

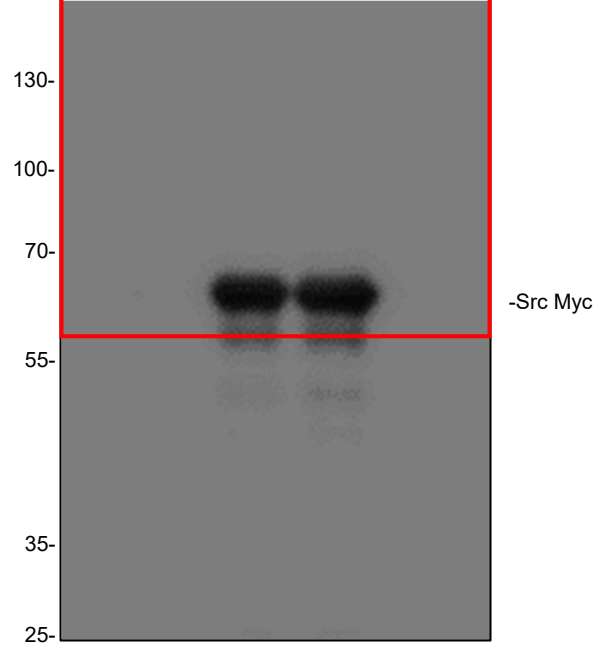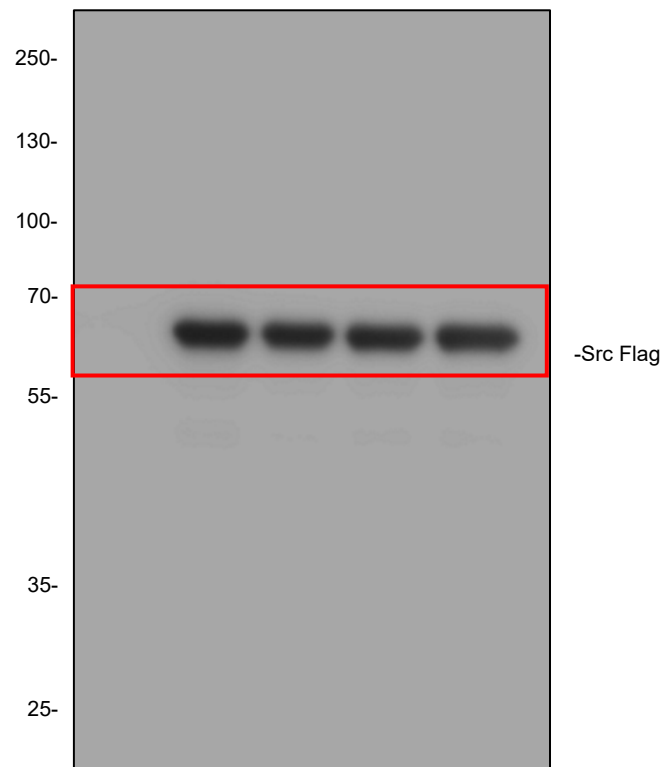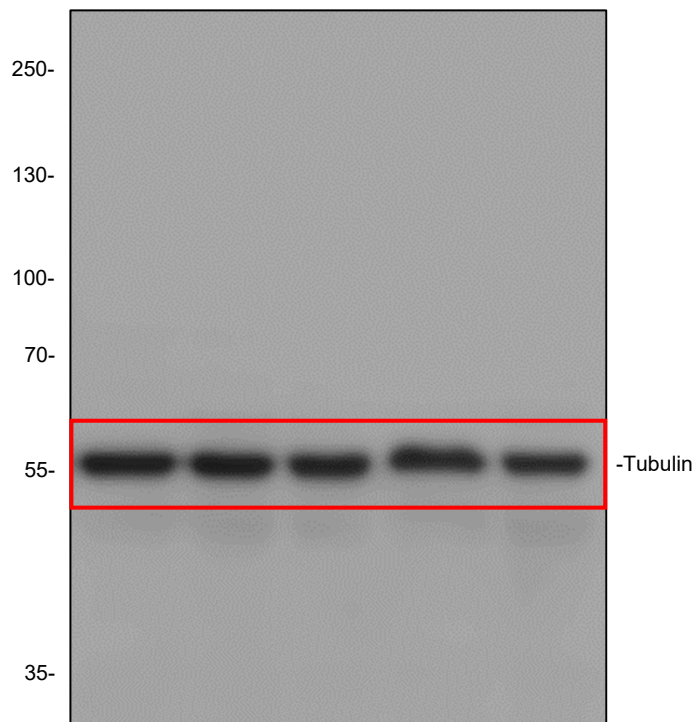

Supplement: Supplementary file 3 [file LSA-2024-03019_SdataFS6.pdf]

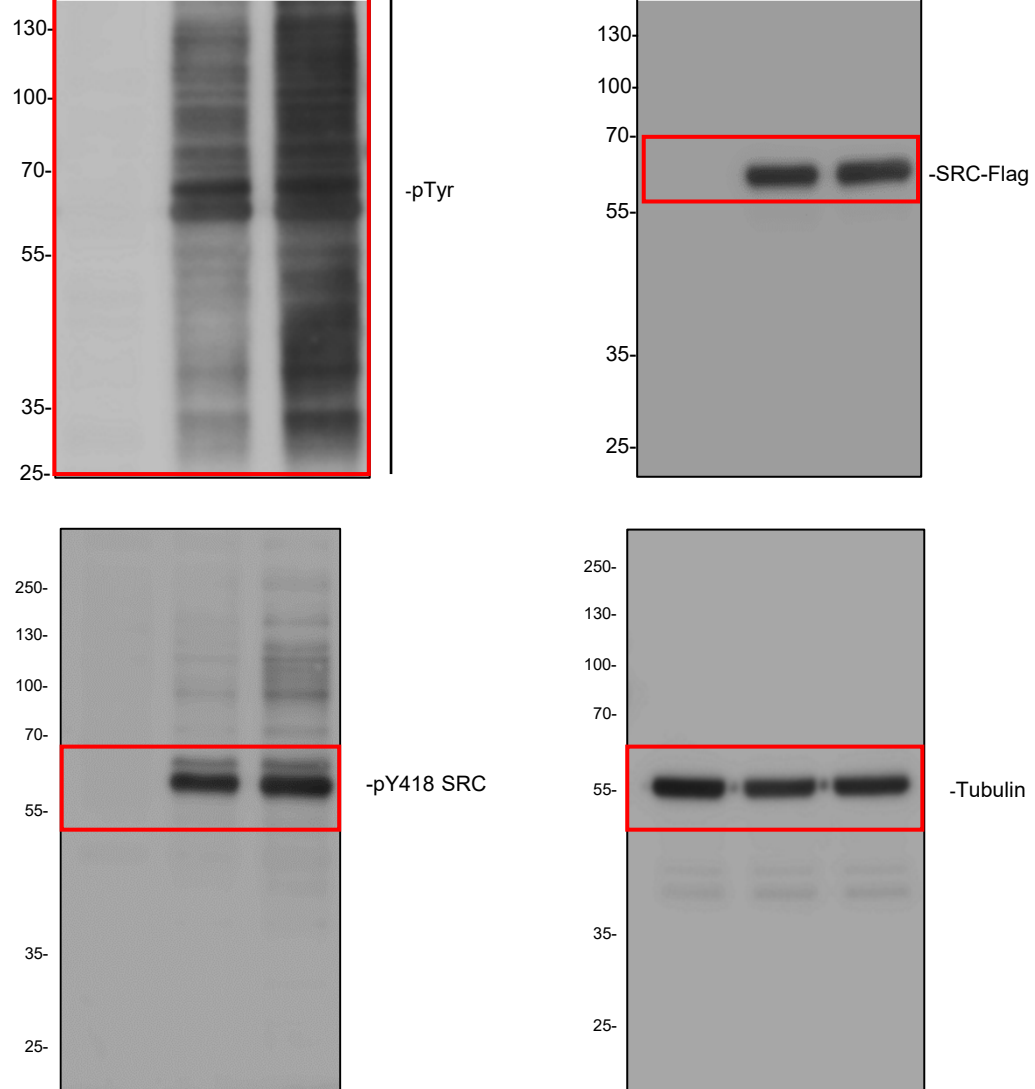

Figure 5 blot B

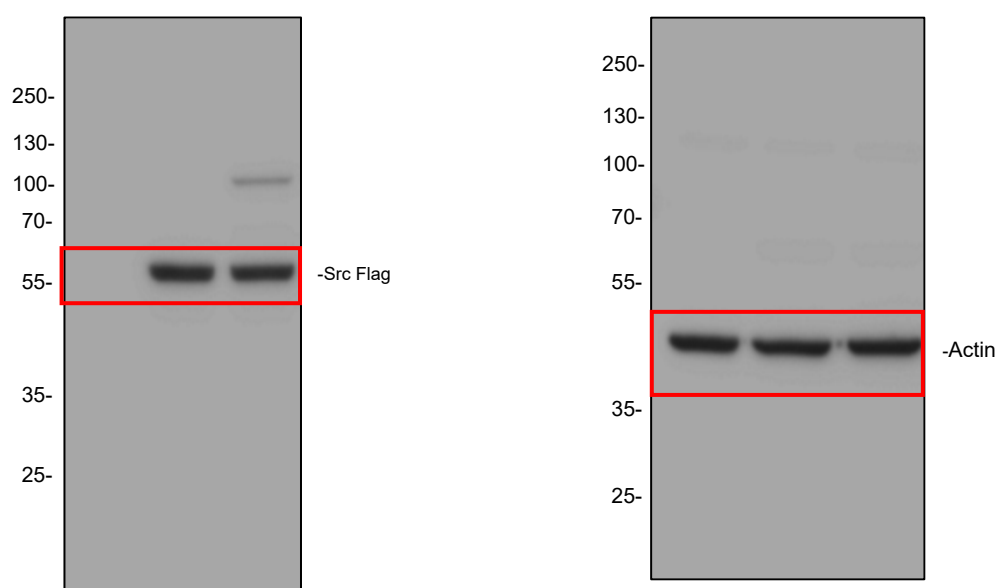

Supplement: Supplementary file 4 [file LSA-2024-03019_SdataF5.pdf]
